# Targeting human SET1/MLL family of proteins

**DOI:** 10.1002/pro.3129

**Published:** 2017-03-06

**Authors:** Masoud Vedadi, Levi Blazer, Mohammad S. Eram, Dalia Barsyte‐Lovejoy, Cheryl H. Arrowsmith, Taraneh Hajian

**Affiliations:** ^1^ Structural Genomics Consortium, University of TorontoTorontoONM5G 1L7; ^2^ Department of Pharmacology and ToxicologyUniversity of TorontoTorontoONM5S 1A8; ^3^ Princess Margaret Cancer Centre and Department of Medical BiophysicsUniversity of TorontoTorontoONM5G 2M9

**Keywords:** methyltransferases, SET1 complexes, OICR‐9429, MM‐401, MI‐2‐2, EPZ004777, EPZ‐5676, SGC0946, mixed‐lineage leukemia 1, leukemogenic rearrangements

## Abstract

The SET1 family of proteins, and in particular MLL1, are essential regulators of transcription and key mediators of normal development and disease. Here, we summarize the detailed characterization of the methyltransferase activity of SET1 complexes and the role of the key subunits, WDR5, RbBP5, ASH2L, and DPY30. We present new data on full kinetic characterization of human MLL1, MLL3, SET1A, and SET1B trimeric, tetrameric, and pentameric complexes to elaborate on substrate specificities and compare our findings with what has been reported before. We also review exciting recent work identifying potent inhibitors of oncogenic MLL1 function through disruption of protein–protein interactions within the MLL1 complex.

**Abbreviations**
AML: acute myeloid leukemia
ALL: acute lymphoid leukemia
ASH2L: absent, small, or homeotic‐like 2
CDK: cyclin dependent kinase
GOF: gain of function
HGNC: HUGO Gene Nomenclature Committee
HMT: histone methyltransferase
MLL: mixed lineage leukemia
PHD: plant homeodomain
RAD: RbBP5–ASH2L–DPY30 complex
RbBP5: RB binding protein 5
WDR5: WD repeat domain 5
WIN: WDR5‐interacting motif
WRA: WDR5–RbBP5–ASH2L complex
WRAD: WDR5–RbBP5–ASH2L–DPY30 complex
SAM: *S*‐adenosylmethionine
SET: Su(var)3‐9, Enhancer of Zeste, Trithorax

## Introduction

**Figure 1:**
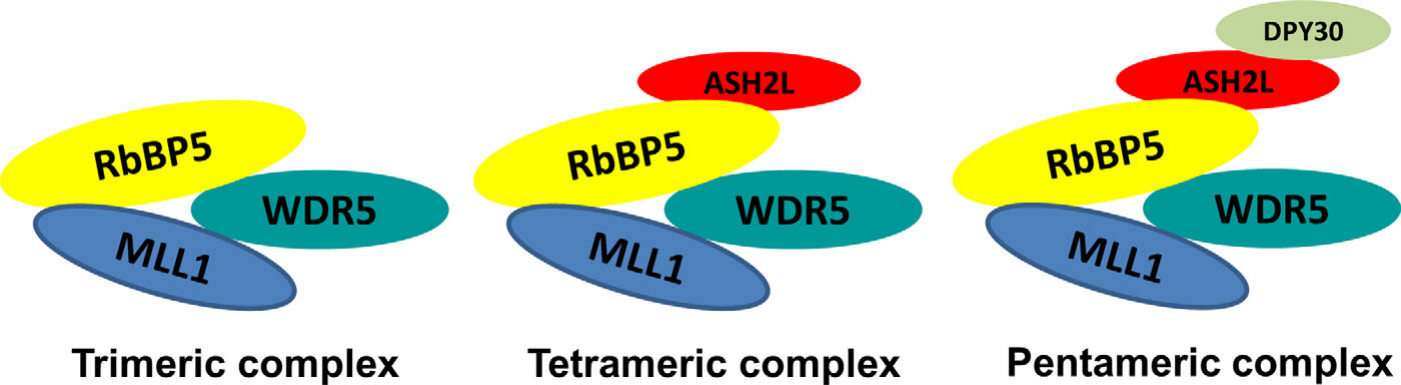
MLL complexes. Trimeric, tetrameric, and pentameric MLL complexes.^106^, ^124^

Chromosomal translocations in acute lymphoid leukemias (ALLs) and acute myeloid leukemias (AMLs) were discovered many years ago.^1^, ^2^, ^3^ Detailed analyses of these leukemogenic rearrangements led to discovery of the involvement of human MLL1 (mixed‐lineage leukemia 1) in disease.^4^, ^5^, ^6^ MLL1 (KMT2A) is a histone 3 lysine 4 (H3K4) methyltransferase with multiple domains including the catalytic domain (MLL‐C; 180 kDa) which forms a complex with WDR5, ASH2L, RbBP5, and DPY30 (Fig. 1) and the N‐terminal domain (MLL1‐N; 320 kDa) that interacts with Menin.^7^, ^8^, ^9^ MLL1 also interacts with other proteins including nuclear cyclophilin33 (Cyp33) and histone deacetylase HDAC1.^10^ MLL1 undergoes several types of rearrangements, all of which have been correlated with leukemogenesis. These include balanced translocations, tandem duplications, and amplification of an otherwise wild‐type form of MLL1. There are also a large number of coding, frameshift, or nonsense mutations in the MLL family of proteins that have been discovered, although the physiological relevance of these are as of yet unknown.^11^ There are more than 100 unique translocations of MLL1 with over 60 translocation partners.^4^, ^5^, ^6^, ^12^, ^13^, ^14^, ^15^, ^16^ While it remains unclear why the MLL1 locus is so exquisitely sensitive to rearrangement, the repertoire of MLL1 translocations that occur in cancer have been well‐studied.^14^, ^17^, ^18^ Regardless of the rearrangement involved, MLL1 translocation‐dependent cancers are highly prone to relapse and require aggressive treatment.^19^, ^20^ Translocations of MLL1 occur in approximately 5% of acute lymphoblastic leukemias (ALL) and 5–10% of acute myeloid leukemia (AML) cases in adults as well as in more than 70% of infant ALL and 35–50% of infant AML patients (reviewed by Chen and Armstrong).^21^ MLL1 translocations also occur in therapy‐related cancers, generally in response to topoisomerase inhibitors (e.g., etoposide).^21^, ^22^, ^23^, ^24^

**Figure 2:**
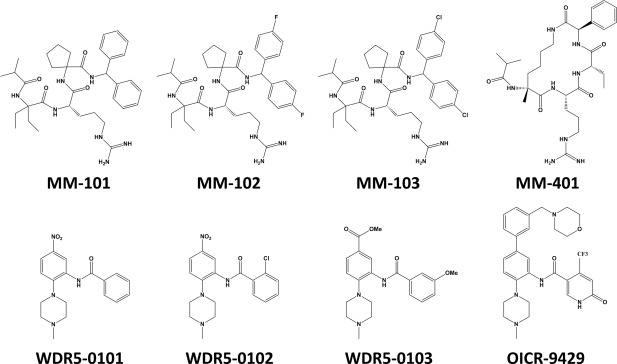
Antagonists of WDR5–MLL interaction. Peptidomimetic antagonists of WDR5–MLL interaction MM‐101, MM‐102, and MM‐103 were designed based on the minimum amino acid (ARA) requirement for WDR5–MLL interaction.^105^ MM‐401 is the follow up cyclic peptidomimetic compound that also disrupt the WDR5–MLL interaction with high potency.^86^ WDR5‐0101 was identified through high throughput screening of 16000 diverse small molecules.^106^ WDR5‐0102 and WDR5‐0103 were commercially available analogues of WDR5‐0101.^106^ OICR‐9429 was synthesized through extensive crystal structure‐guided chemistry.^92^

**Figure 3:**
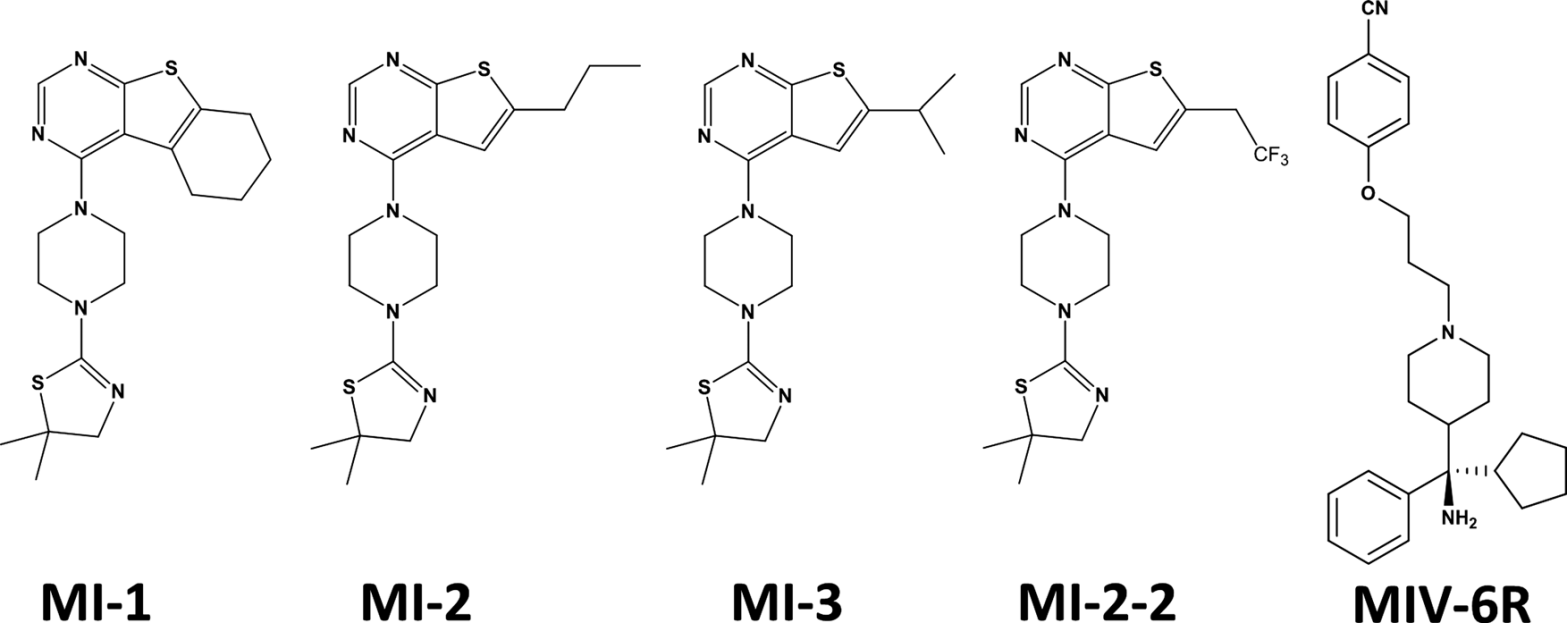
Antagonists of Menin–MLL interaction. MI‐1, MI‐2, and MI‐3 were identified through high throughput screening of a library of 49,000 small molecules using FP assay.^102^ MI‐2‐2 was designed and synthesized through structure‐based follow up chemistry by replacing *n*‐propyl with trifluoroethyl group in MI‐2.^111^ MIV‐6R was designed and synthesized through structure‐guided chemistry following identifying hydroxy‐ and aminomethylpiperidine screening hit compounds.^112^

MLL1‐rearranged leukemia has been shown to be associated with high expression of the homeobox (HoxA) cluster genes, transcription factors that specify cell identity during hematopoiesis and favour immortalization of leukemic cells.^25^ MLL1 fusions cause persistent activation of HoxA9 and its cofactor MEIS1 that are essential for sustaining the leukemic phenotype.^26^ Globally MLL1‐fusions preferentially regulate a subset of the genes that are wild‐type MLL targets and significantly increase the transcription of developmentally important genes involved in the disease phenotype.^27^, ^28^ Wild‐type MLL1 is essential for hematopoiesis and neurogenesis, driving the gene expression programs that regulate stem cell function.^29^, ^30^ In cancer, these transcriptional programs are hijacked for cancer growth and angiogenesis and are driven, at least in part, by the ability of MLL1 to promote expression of MYC and cyclin‐dependent kinases.^31^, ^32^, ^33^

The breakpoint of most MLL1 translocations occurs just downstream of the CXXC domain, leading to the deletion of the PHD and catalytic SET domains.^34^ Loss of normal catalytic activity by the fusion protein necessitates the maintenance of a single wild‐type allele of MLL1 for leukemogenesis.^35^, ^36^ However, this effect is not dependent solely on the histone methyltransferase (HMT) activity of MLL1,^37^ as MLL1 fusion proteins also require wild‐type MLL1 prebinding to the HoxA9 locus for stable association.^38^ Taken together, this suggests that wild‐type MLL1 activity is required for the full transformative capacity of MLL fusion proteins and that targeting the catalytic activity of MLL1 may be an attractive mechanism for cancer chemotherapy.

MLL1 fusion proteins are generally considered gain‐of‐function (GOF) changes with potent transcriptional regulatory abilities. For example, fusion of MLL with ENL or AF9 leads to recruitment of the SWI/SNF complex to dysregulate the expression of oncogenic genes including HoxA7.^39^, ^40^ Blocking the ability of MLL1 fusion proteins to properly localize to the promoters of growth‐stimulating genes is another area that is being actively targeted for pharmacological intervention. The most direct method to inhibit MLL1 recruitment is to disrupt the protein–protein interactions required for complex formation. MLL1 is a member of a large and dynamic protein complex that requires the presence of Menin to regulate Hox and CDK gene expression.^32^, ^41^ Genetic deletion of Menin is able to diminish the H3K4 methylation levels at Hox loci more effectively than genetic deletion of MLL1.^42^ While the limited effect of MLL1 ablation on H3K4 methylation may be due to functional redundancy between MLL family members, it remains clear that association of MLL1 with Menin is required for proper H3K4 methylation patterns at Hox loci. MLL1 fusion proteins directly interact with Menin, but are unable to bind other members of the MLL1 complex.^41^ This MLL fusion–Menin interaction is essential for leukemogenic transformation.^7^ Disrupting this protein–protein interaction is another approach to target the function of MLL fusion proteins. However, loss‐of‐function mutations in Menin lead to multiple endocrine neoplasia type I,^43^, ^44^ so it is important to ensure that small molecule inhibitors of the MLL1–Menin protein–protein interaction do not globally affect Menin activity.

Other MLL family members have also been implicated in disease.^11^, ^45^ The human SET1 family of proteins includes MLL1 (KMT2A), MLL2 (KMT2D), MLL3 (KMT2C), MLL4 (KMT2B), SET1A (KMT2F), and SET1B (KMT2G). Here we included the HUGO Gene Nomenclature Committee (HGNC) approved gene symbols (http://www.genenames.org/) because there has been major confusion in the gene nomenclature for MLL2 and MLL4 in the literature^46^ with both names interchangeably being used for two different genes. Mouse versus human nomenclature used in related reports may also add to confusion. This results in further confusion when the gene names and numbers are not indicated in publications. Therefore we suggest that readers pay special attention to the gene names, symbols and chromosome locations when reading the related publications if provided (MLL2: KMT2D; Chromosome 12q13.12; Gene MIM 602113; http://omim.org/entry/602113 and MLL4: KMT2B; Chromosome 19q13.12; Gene MIM 606834; http://omim.org/entry/606834 for human proteins).

A large number of somatic mutations have been identified from a panel of over 3,000 samples representing 12 tumor types for MLL2, MLL3, or MLL4.^47^ While the relevance of the majority of these mutations are as of yet unknown, mutations in MLL2 are linked to non‐Hodgkins lymphoma,^48^ pancreatic cancer,^49^ and medulloblastoma,^50^, ^51^, ^52^ as well as impaired glucose tolerance and insulin resistance.^53^ Similarly, MLL3 mutations are implicated in colorectal,^54^ pancreatic,^49^ nasopharyngeal,^55^ medulloblastoma,^51^ and other cancers.^11^, ^56^, ^57^ Recent studies have shown that some of these MLL3 mutants are located in the methyltransferase active site and dramatically alter enzymatic activity^56^ and loss of activity may contribute to progression of AML.^58^ Such loss of function mutations makes the protein a less desirable target for drug discovery in comparison to gain of function mutations. MLL4 mutations or translocations have been implicated in spindle cell sarcoma^59^ and hepatocellular carcinoma.^60^ While the major pathological mechanisms underlying most of these mutations are not fully understood, it is clear that mutations and rearrangements of the MLL family proteins are important in the initiation and maintenance of broad range of cancers. The reader who is further interested in the intricacies of MLL disease mechanisms will find the following references particularly enlightening.^11^, ^17^, ^61^, ^62^, ^63^, ^64^ Extensive implications of SET1 family of proteins in diseases validate these proteins as potential drug targets. In cases such as MLL1, finding potent and selective inhibitors would help further investigate their involvement in diseases and discovery of therapeutics. This necessitates full kinetic characterization of these protein complexes, and optimizing assays for high throughput screening.

## Methyltransferase activity of SET1 family of proteins

The first biochemical reconstitution of mammalian MLL1 four subunit complex revealed that the catalytic SET domain of the wild‐type MLL1 is only significantly active in the presence of structural components of the MLL1 complex, RbBP5, ASH2L, and WDR5^65^ (Fig. 1). Although di‐ (H3K4me2) and trimethylation (H3K4me3) of H3K4 was observed with MLL1 complex, dimethylation was much more pronounced indicating higher catalytic efficiency for mono‐ and dimethylation than trimethylation. Presence of WDR5 and RbBP5, but not ASH2L, was shown to be essential for MLL1 complex formation. In particular, WDR5 is absolutely essential for MLL1 complex integrity and activity.^65^ Human MLL1 (residues 3745–3969) in complex with WDR5, RbBP5 and ASH2L with (MWRAD) or without DPY30 (MWRA) were also reported to be a better monomethylase (∼1 h^−1^) than dimethylase (∼0.2 h^−1^).^66^ Accumulation of monomethylated product also suggested a nonprocessive mechanism. In addition to MLL1, the SET domains of MLL2, MLL4, SET1A and SET1B displayed negligible enzymatic activity in the absence of the WDR5–RbBP5–ASH2L complex. MLL3, however, actively methylated histone H3 in the absence of the core complex subunits.^67^ WDR5 was reported to bind tightly to MLL1 (*K*_D_ value of 120 n*M*) and with less affinity to RbBP5 (*K*_D_ of 2400 n*M*) within the core complex.^66^ Interaction of ASH2L with the core complex appeared to be mediated by RbBP5.^65^, ^66^ A heterodimer of ASH2L and RbBP5 (*K*_D_ of 800 n*M* 66) has been reported to also have intrinsic HMT activity.^68^ Lack of trimethylation was also noted in the absence of ASH2L and RbBP5.^65^ Reduction in ASH2L using RNA interference also led to loss of H3K4 trimethylation with no detectable effect on H3K4 mono‐ or dimethylation levels.^69^ WDR5, RbBP5 and ASH2L were reported to form a stable complex in the absence of catalytic domain of MLL1.^70^ Patel and colleagues reconstituted human WDR5, RbBP5, ASH2L, and DPY30 (WRAD) complex *in vitro* and using micromolar concentrations of WRAD they were able to show basal levels of HMT activity for the WRAD (*k*_cat_: 30 h^−1^) and WRA (*k*_cat_: 70 h^−1^) complexes in the absence of catalytic subunit of MLL1 (note that the values are from correction of the initial values in Table 1 by authors).^70^ Shinsky and colleagues report that absence of RbBP5 or ASH2L subunits completely abolish the stimulatory effect of WRAD on activity of SET1 family of proteins.^71^ However, WDR5 may not be essential for MLL2, MLL3, MLL4 and SET1B activities.^71^ They also did not observe any significant change upon addition of DPY30. However, recently the upregulation of DPY30 expression in gastric cancer cell lines and patients' tissues has been reported. A decrease in proliferation, migration, and invasion of gastric cancer cells upon DPY30 knockdown by siRNA was also observed.^72^ DPY30 has been reported to regulate proliferation and differentiation of hematopoietic progenitor cells by regulating the expression of genes critical for cell proliferation.^73^ These observations suggest that DPY30 plays a critical role in cells.

**Table 1:** Kinetic Characterization of SET1 Family of Proteins

Enzyme	Complex	SAM			H3K4me0			H3K4me1			H3K4me2		
		*K*_m_ (µ*M*)	*k*_cat_ (h^−1^)	*k*_cat_/*K*_m_	*K*_m_ (µ*M*)	*k*_cat_ (h^−1^)	*k*_cat_/*K*_m_	*K*_m_ (µ*M*)	*k*_cat_ (h^−1^)	*k*_cat_/*K*_m_	*K*_m_ (µ*M*)	*k*_cat_ (h^−1^)	*k*_cat_/*K*_m_
MLL1	Trimeric	8 ± 2	14 ± 1	1.8	6 ± 1	15 ± 7	2.5	3 ± 1	5 ± 0.7	1.7	NA	<1	NA
	Tetrameric	2 ± 0.3	29 ± 4	14.5	9 ± 4	37 ± 6	4.1	3 ± 0.1	20 ± 3	6.7	NA	<2	NA
	Pentameric	0.6 ± 0.01	13 ± 2	21.7	2 ± 0.1	14 ± 2	7.0	0.5 ± 0.1	9 ± 1.5	18.0	NA	<2	NA
MLL3	Trimeric	170 ± 7	75 ± 13	0.4	23 ± 3	60 ± 15	2.6	NA	<2	NA	NA	<1	NA
	Tetrameric	80 ± 7	1128 ± 121	14.1	17 ± 3	1100 ± 130	64.7	NA	NA	NA	NA	∼6	NA
	Pentameric	51 ± 9	1200 ± 160	23.5	8 ± 1	1200 ± 200	150.0	NA	NA	NA	NA	<2	NA
SET1A	Trimeric	210 ± 20	25 ± 1	0.1	47 ± 3	28 ± 2	0.6	NA	<1	NA	NA	<1	NA
	Tetrameric	290 ± 60	72 ± 25	0.2	24 ± 10	56 ± 14	2.3	8 ± 1	12 ± 5	1.5	NA	∼4	NA
	Pentameric	170 ± 60	100 ± 30	0.6	17 ± 2	94 ± 17	5.5	17 ± 3	30 ± 9	1.8	NA	∼6	NA
SET1B	Trimeric	>50	> 15	NA	>10	>15	NA	>10	<2	NA	NA	<1	NA
	Tetrameric	3 ± 2	7 ± 0.5	2.3	3 ± 0.6	9 ± 0.4	3.0	5 ± 3	4 ± 1	0.8	NA	<1	NA
	Pentameric	3.4 ± 0.04	13 ± 0.5	3.8	3 ± 1	19 ± 8	6.3	3 ± 1	6 ± 0.8	2.0	NA	<1	NA

All values in this table are apparent values that have been determined under the experimental conditions used in this study.

WDR5 interacts with MLL1, through a conserved arginine containing WIN (WDR5 Interacting) motif,^74^, ^75^ and with histone H3 via di‐ and trimethylated K4 either as an isolated histone or in the context of an H3K4‐dimethylated nucleosome.^76^ WDR5 is required for binding of the methyltransferase complex to the K4‐dimethylated H3 tail as well as for global H3K4 trimethylation and Hox gene activation in human cells.^76^ WDR5 binds to the H3 N‐terminal tail in a manner that leaves the K4 residue fully exposed; a conformation that is ideal for presenting the substrate for methylation.^77^, ^78^ The WIN motif is conserved in human SET1 family members (**G**(S/C/A)**AR**(A/S)**E;** conserved amino acids are in bold).^79^ However, WDR5 binding affinity for peptides derived from these sequences differ, with K_D_ values of 2.8 µ*M* for MLL1 peptide and 75, 54, 88, 541, and 103 n*M* for MLL2, MLL3, MLL4, SET1A, and SET1B respectively.^79^ The presence of WIN peptides was shown to inhibit the HMT activity of SET1 family of proteins,^74^, ^79^ perhaps through disruption of the complex. The conserved arginine residue (Arg‐3765 in MLL1) has been shown to be essential for assembly of the complex and MLL1‐mediated H3K4 dimethylation.^74^ Mutation of this arginine to alanine resulted in disruption of the core complex formation.^79^

Shinsky and Cosgrove have reported that the RbBP5‐ASH2L (RA) heterodimer interacts with MLL3 SET domain in the absence of WDR5.^80^ MLL3 only monomethylated H3K4, showing no di‐ or trimethylation activity.^80^ In contrast to MLL1, the HMT activity of MLL3 was reported to be about 100‐fold higher in the absence of WDR5 than in complex with RbBP5, ASH2L, and DPY30 (3.96 ± 0.22 h^−1^) and was inhibited by the presence of WDR5.^80^ These results were obtained from fluorograms and single‐turnover enzymatic assays using micromolar enzyme concentrations monitored by mass spectrometry. The inhibitory effect of WDR5 on MLL3 activity is particularly interesting as WDR5 binds to MLL3 WIN derived peptide with the highest affinity of any WIN motif from a SET1 family member^79^ and binds to MLL3 through arginine 4710 forming an stable 1:1 complex.^80^ Furthermore, Zhang and colleagues previously reported that the core complex subunits stimulate the HMT activities of MLL2, MLL3, MLL4, SET1A, and SET1B and in the absence of WDR5 the activities of SET1A, MLL3, and MLL4 core complexes decrease by twofold. This effect was also observed for SET1B and MLL2. The authors noted an HMT activity for MLL3 in the absence of the core complex suggesting a role for MLL3 independent of the WDR5–RbBP5–ASH2L complex.^67^ Through structural analysis they also suggested that binding of the WIN motifs is achieved by the plasticity of WDR5 peptidylarginine‐binding cleft allowing the C‐terminal ends of the WIN motifs to have structurally divergent conformations.^67^ Structural aspects of MLL complexes have been previously reviewed.^81^, ^82^

An activity of 30 h^−1^ has been observed for MLL4 SET domain that was significantly increased in the presence of WRAD (159 h^−1^) as measured by an HPLC‐based assay that separated ^3^H‐labeled peptides.^83^ Based on structural interpretations, this higher intrinsic activity was attributed to possible hydrogen bonds between residues of the post‐SET loop (e.g., Asp5519) with residues from SET‐I region which may stabilize an active MLL4 SET domain conformation.^83^ Using mass spectrometry and single turnover assays, only monomethylation was observed after a 60 min reaction, but dimethylation was observed if the reaction was allowed to proceed overnight. However, in complex with WRAD, di‐ and trimethylated species were detected. Although there is a clear difference in levels of activities of MLL4 and MLL1 SET domains in the absence of the complex components, both show similar levels of activities when in WRAD complex. This resulted in speculation that the presence of WRAD may induce SET‐I movements which help forming a more catalytically efficient active site conformation.^83^

## Available methyltransferase assays

Many of the initial discoveries surrounding the HMT activity of SET1 family proteins utilized radiometric assays to demonstrate enzymatic activity. These assays are based upon the transfer of a radiolabeled (generally ^3^H) methyl group from the cofactor *S*‐adenosyl‐methionine (SAM) to a substrate lysine. The reactants are then separated using SDS‐PAGE and incorporated radioactivity is measured using autoradiography.^65^, ^71^, ^84^, ^85^, ^86^ While this approach is invaluable for the initial discovery and characterization of methyltransferases, its low‐throughput methodology and limited dynamic range renders it unsuitable for compound screening and discovery of chemical probes (potent and selective inhibitors or antagonists). To address this issue, we and others have developed a series of assays that accommodate the requirements of medium‐ or high‐throughput screening.^86^, ^87^, ^88^, ^89^, ^90^, ^91^ In addition to facilitating the discovery of chemical probes targeting a number of methyltransferases,^89^, ^92^, ^93^ the development of these assays has also provided a means to more thoroughly characterize the biochemical activity of many HMTs, including the SET1 family of methyltransferases.

The current gold‐standard assay for measuring methyltransferase activity is an adaptation of the original radiometric assay. In this assay format, transfer of a tritiated methyl group from the cofactor SAM to the lysine substrate (peptide, histone, nucleosome) is measured by separating the labeled reaction product from the free [^3^H]SAM and quantifying the incorporated radioactivity via scintillation counting. There are several separation techniques that are suitable for the needs of compound screening. For core histone and nucleosome substrates, the easiest separation method is to precipitate the substrate using trichloroacetic acid (10%) followed by capture on a glass fiber filter. Residual SAM is removed by repeated washing steps. This filter‐based methodology is amenable to 96‐ and 384‐well format,^94^, ^95^, ^96^ however the throughput is limited by the necessity of extensive washing steps. An alternative method is to use an affinity‐capture method to separate the radiolabeled substrate from the free [^3^H]SAM. Biotinylated peptide substrates can be immobilized using biotin‐capture membranes (e.g., SAM2^©^ Biotin Capture Membrane, Promega) for standard liquid scintillation analysis. These membranes provide a high binding capacity and are suitable for characterization of low‐turnover enzymes, but, like autoradiography, they have the lowest‐throughput of their respective class of assay technology. However, this is a more reliable method for kinetic characterization of methyltransferases.^96^, ^97^

A higher throughput option within the affinity capture methods is based upon the use of scintillation proximity plates to measure radiolabel incorporation without requiring the removal of [^3^H]SAM. The wells of these plates are coated with streptavidin and have a thin layer of a solid‐phase scintillant on the walls of the plate itself. A biotinylated substrate is drawn into close physical proximity with the walls of the SPA plate via the biotin–streptavidin interaction and it is only at these close ranges that the radiolabel is detected by the scintillant. This assay format therefore requires no wash steps to remove unincorporated radiolabel, making it particularly well‐suited to the needs of high‐throughput screening.^93^, ^98^ Fluorescence‐based methods such as the SAH hydrolase‐coupled assay^99^ have also been optimized for high throughput screening of HMTs^90^ and successfully used to identify potent inhibitors.^100^ However, this method requires de‐coupling steps and may have higher false positive rates than radioactivity‐based high throughput screening methods. Chemiluminescence‐based method have been optimized for screening methyltransferases such as G9a,^91^ however this assay also requires counter screening to filter out possible false positives. Microfluidic capillary electrophoresis assays are also useful for characterization of HMTs.^101^ Lower throughput methods or those requiring the employment of expensive instrumentation such as mass spectrometry‐ or NMR‐based methods are possibly more sensitive and more useful for hit confirmation.^71^, ^102^

## Available binding assays

In addition to methyltransferase activity assays, a number of binding assays have been developed for this family of proteins. Fluorescence polarization‐based assays have been developed to assess binding of peptides to WDR5.^103^ It was shown that WDR5 is essential for HMT activity of MLL1^65^ and the minimal MLL1 sequence required for interaction of WDR5 with MLL1 was mapped to “ARA” with *K*_i_ value of 120 n*M* for “Ac‐ARA”. In comparison the *K*_i_ value for WIN peptide (Ac‐GSARAEVHLRKS) binding to WDR5 was 160 n*M* 103 (WDR5 and MLL1 form a stable complex in solution with a *K*_D_ value of 120 n*M* 74). Interaction of WDR5 with “ART” of histone H3 tail was tighter with a *K*_i_ value of 20 n*M*.^103^ Taking advantage of available amino acid sequence of WIN motif of MLL1, we also developed a peptide (WIN: GSARAEVHLRKS) displacement assay to screen in a 384‐well format for compounds that bind to WDR5 and inactivate MLL1 by disrupting MLL1–WDR5–RbBP5 complex.^90^ In these assays, binding of the fluorescein labeled peptide to WDR5 increases the fluorescence polarization (FP) signal. Displacement of the labelled peptide by potential ligands can therefore be detected by monitoring a decrease in signal. This assay was optimized for screening in 384‐well format with a Z′‐factor of 0.6.^90^ Very recently, we also developed a SAM displacement assay for MLL1.^104^ In this assay, a small molecule fluorescent ligand (FL‐NAH) that is able to bind to the SAM binding site of MLL1 in a manner independent of the associated complex members was used to develop a fluorescence polarization‐based SAM displacement assay in 384‐well format. FL‐NAH binds to MLL1 SET domain in the absence of associated complex members and competes with SAM, SAH, and the fungal metabolite sinefungin, but not with a peptide corresponding to residues 1–25 of histone H3. This assay enables screening for SAM‐competitive MLL1 inhibitors without requiring the use of trimeric or higher order MLL1 complexes, significantly reducing screening time and cost.^104^

## Kinetic characterization of human SET1 family of proteins

One of the questions that has already been proposed and investigated is whether the components of the SET1 complexes affect the ability of the catalytic subunit to mono‐, di‐ or trimethylate.^65^, ^66^ To further investigate this and also fully characterize the kinetics of HMT activity of SET1 family members and compare their substrate specificities, we reconstituted human MLL1 (3745–3969), MLL3 (4706–4911), SET1A (1491–1707), and SET1B (1815–2037) trimeric (MWR), tetrameric (MWRA) and pentameric (MWRAD) complexes (W; 1–334, R; 1–538, A; 1–628, D; 1–99) as described in the Supporting Information Materials and Methods. Using histone H3 peptides with various H3K4 methylation states (H3K4me0, H3K4me1, and H3K4me2) as substrate and Scintillation Proximity Assay (SPA) as well as biotin‐capture membranes, we determined the kinetic parameters (Michaelis–Menten kinetics) for each enzyme in all three complex forms (Table 1, Supporting Information Figs. S1–5). The experiments were performed under linear initial velocities (Supporting Information Fig. S6) using optimized assay conditions (Supporting Information Table SI and Fig. S7). Trimethylation of H3K4 by tri‐, tetra‐ or pentameric MLL1 complexes was not accurately measurable. However, MLL1 ability to mono‐ or dimethylate increased with higher complexes (M_1_WRAD > M_1_WRA > M_1_WR) reaching catalytic efficiencies (*k*_cat_/*K*_m_) of 7 and 18 µ*M* ^–^ 1 h^−1^, respectively with pentameric complex. This is consistent with previous reports suggesting MLL1 only mono‐ and dimethylates H3K4 through a distributive mechanism.^66^, ^71^ However, the level of MLL1 complex activity in our hands was more than 20–40 times higher than values previously reported.^66^, ^71^ This may reflect our assay optimization and using Michaelis–Menten kinetics. Note that the presence of salt, and in particular NaCl, in the assay mixture significantly reduces the activity of SET1 family of proteins (Supporting Information Fig. S7). Lower turnover rates previously reported may be the result of using high concentrations of salt in assay buffers.^66^, ^71^ MLL3 was the most active monomethyltransferase of the four SET1 family members we characterized with *k*_cat_ value of 1200 ± 200 h^−1^ for pentameric complex. Catalytic efficiency of pentameric complex was more than 50‐fold higher than that for trimeric complex. This is consistent with reports that MLL3 core complex is predominantly a monomethylase.^71^ We were not able to reliably determine any dimethylation activity for MLL3. Interestingly, tetrameric and pentameric MLL3 complexes showed some residual trimethylation activities (2–6 h^−1^) when H3K4me2 was used as substrate. The trimeric complex of SET1A or SET1B prepared through final step of size exclusion purification showed no measurable activity. However, increasing the ratio of SET1A SET domain to this trimeric complex preparation (3:1) resulted in a significant level of monomethyltransferase activity [*k*_cat_ of 28 ± 2 h^−1^; Supporting Information Fig. S1(A)] but not di‐ or trimethylation. No further stimulation was observed when 4:1 ratio was tested. A similar pattern was observed for SET1B trimeric complex [Supporting Information Fig. S1(B)] with a *k*_cat_ of about 15 h^−1^. Similarly no significant di‐ or trimethylation was observed for trimeric complex. Tetrameric and pentameric SET1A were both better monomethyltransferases than dimethyltransferase and showed significant but low (*k*_cat_ of 4–6 h^−1^) levels of trimethylase activities. SET1B appeared to be about fivefold less active than SET1A. Trimethyltransferase activity of SET1A and SET1B is consistent with previous reports.^71^ All MLL members were significantly more efficient at utilizing peptide substrates with unmethylated or monomethylated rather than dimethylated H3K4.^71^, ^86^

## Discovery of Inhibitors of SET1 Family of Proteins

### Antagonists of WDR5–MLL interaction (Fig. 2)

The broad diversity in SET1 family expression patterns and rearrangements in cancer makes them intriguing drug targets. Interestingly, even though the MLL1 fusion protein is potently oncogenic, it does not contain an active catalytic domain but requires the maintenance of a wild‐type allele for leukemogenesis.^36^ Therefore, inhibition of wild‐type MLL1 HMT activity could be a valid approach to discover novel therapeutics targeting MLL‐rearranged leukemias. As WDR5 is essential for the integrity and HMT activity of MLL1 complex,^65^ compounds that compete with the WDR5–MLL interaction could potentially inhibit the MLL HMT activity by disruption of the complex. To this end, Karatas and colleagues designed a series of peptidomimetic antagonists of this interaction based on the minimum amino acid (ARA) requirement for WDR5–MLL1 interaction. These efforts resulted in discovery of MM‐101, MM‐102, and MM‐103 (‐H, ‐F, and Cl substitutions, respectively) with binding IC_50_ values of 2.9, 2.4, and 4.5 n*M*, respectively. Amongst the compounds tested for inhibition of the HMT activity of tetrameric MLL1 complex, MM‐102 was the most potent with an IC_50_ value of 400 n*M*.^105^ Consequently, the authors tested the effect of this compound on expression of HoxA9 and Meis‐1 that are highly expressed during leukemogenesis. MM‐102 reduced the expression of HoxA9 in myeloblasts with an IC_50_ value of around 25 µ*M*, but had a much weaker effect on expression of Meis‐1 (∼40% at 50 µ*M*). The authors synthesized a negative control that had no effect on HoxA9 or Meis‐1 expression by substituting the l‐arginine in MM‐102 with d‐arginine.^105^ Further development of this series led to the cyclic peptidomimetic compound MM‐401, which maintained the high WDR5 binding affinity (half maximum displacement (*K*_disp_) of 0.9 n*M*), and the ability to inhibit HMT activity of MLL1 complex (IC_50_ value of 320 n*M*).^86^ MM‐401 had no effect on the activity of MLL2, MLL3, MLL4, and SET1A or their methylation‐state specificities which was attributed to the dispensability of WDR5 for activity of these proteins.^86^ At 20 µ*M*, MM‐401 had no effect on global H3K4 methylation but reduced the H3K4me2 and H3K4me3 across 5′ HoxA loci in MLL–AF9 cells after 48 h, and reduced the expression levels of these genes. It was reported that MM‐401 specifically caused cell death and differentiation in MLL1‐AF9, MLL1‐ENL, MLL1‐AF1 mouse models of leukemia [half maximum growth inhibition (GI_50_) of about 10 µ*M*] without affecting normal bone marrow progenitor cells. MM‐401 also inhibited the growth of the blast cells isolated from AML patients with MLL1 rearrangements.^86^

A similar approach was taken to discover small molecule antagonists of the WDR5–MLL1 interaction, by screening a library of 16,000 diverse small molecules by FP‐based peptide displacement assay resulting in the discovery of WDR5‐0101 with *K*_disp_ value of 12 ± 1 µ*M*.^106^ Two commercially available analogs of WDR5‐0101, WDR5‐0102, and WDR5‐0103 were also identified with *K*_disp_ values of 11 ± 1 and 3 ± 0.1 µ*M*, respectively. A *K*_D_ value of 450 n*M* was determined by isothermal calorimetry for WDR5‐0103 binding to WDR5.^106^ Structure guided optimization of WDR5‐0102 resulted in identification of compound **47** with *K*_disp_ of 300 n*M*.^107^ Through an extensive structure‐guided medicinal chemistry effort the potency and cellular activity of this series was improved, yielding OICR‐9429 (*K*_disp_ of 64 ± 4 n*M*; *K*_D_ of 93 ± 28 n*M*) which also binds to WDR5 in the MLL1 WIN motif‐binding pocket.^92^ OICR‐9429 was highly selective for WDR5 with no binding to or inhibition of a panel of more than 250 human methyltransferases, WD40 and histone reader domains, human kinases, G protein–coupled receptor, ion channel, and transporter drug targets. OICR‐9429 reduced the amount of endogenous MLL1 (IC_50_: 223 n*M*) and RbBP5 (IC_50_: 458 n*M*) that co‐immunoprecipitated with exogenously expressed Flag‐tagged WDR5 in a dose‐dependent manner.^92^

OICR‐9429 was used to probe the biology associated with antagonizing WDR5–MLL1 interactions in two systems in which oncogenic transcription factors drive cell growth in a WDR5–MLL dependent manner. In the first case, C/EBPα is a transcription factor that regulates myeloid gene expression in the hematopoietic system and its deficiency leads to a complete block of terminal myeloid differentiation at the pregranulocyte/monocyte‐cell stage.^108^ Frameshifts in the N‐terminal part of the C/EBPα coding sequence which results in expression of a shorter C/EBPα (p30) was reported in AML patients.^109^ p30 interacts with WDR5, colocalizes with H3K4me3 and mediates myeloid differentiation block in a WDR5‐dependent manner. OICR‐9429 inhibited proliferation and induced differentiation in p30‐expressing cells in a mouse model of AML.^92^ OICR‐9429 (5 µ*M*) caused a significant decrease in viability in the majority of AML patient‐derived cells with mutations in the N‐terminal part of the CEBPA gene (mean viability of 53%), with no effect on those lacking the mutations.^92^ In the second case, GOF p53 mutant binds to the transcription factor ETS2 and activates MLL1 and MLL2 genes as well as histone acetyltransferase MOZ resulting in slight changes in genome‐wide increases of histone methylation and acetylation and target gene specific changes in H3K4me3. These modifications activated specific gene expression programs and caused an increase in the proliferation of cancer cells. OICR‐9429 specifically inhibited cell proliferation of GOF p53 mouse embryonic fibroblasts (MEFs), but not when GOF p53 is reduced or in p53 null MEFs. Dose‐dependent inhibition by OICR‐9429 of GOF p53 Li‐Fraumeni Syndrome (LFS) cell growth was also observed, with little effect on p53 null LFS cells.^110^

Together these studies demonstrate the potential therapeutic value of compounds that disrupt the WDR5–MLL1 interaction. Importantly, due to the differential dependence of the SET1 family members on WDR5, this strategy is an alternative to directly targeting the catalytic activity of individual members of the SET1 family, which may be difficult to achieve.

### Antagonists of Menin–MLL interaction (Fig. 3)

Oncogenic MLL1 fusion proteins retain the ability to stably associate with Menin that is required for the initiation of MLL‐mediated leukemogenesis.^7^ Disruption of this interaction is also a viable approach for MLL1‐targeted drug discovery. Menin binds to MLL1 with a *K*_D_ value of 10 n*M* through two Menin‐binding motifs (MBM1 and MBM2) with MBM1 being the high affinity binding motif (residues 4–15).^8^ The first small molecule antagonist of Menin–MLL1 interaction (MI‐1, a thienopyrimidine) was identified through screening 49,000 compounds using a fluorescence polarization‐based peptide displacement assay with IC_50_ (*K*_disp_) value of 1.9 µ*M*.^102^ Follow‐up chemistry on MI‐1 resulted in identifying MI‐2 and MI‐3 with *K*_disp_ values of 446 and 648 n*M* (ITC *K*_D_ values of 158 and 201 n*M*), respectively.^102^ Grembecka and colleagues showed that MI‐2 and MI‐3 at concentrations as low as 12.5 µ*M* efficiently disrupt the Menin–MLL1–AF9 complex in HEK293 cells without affecting the amount of expression of Menin and MLL1–AF9.^102^ These compounds induced down‐regulation of HoxA9 and Meis‐1 expression, inhibited the transforming properties of MLL1–AF9 fusion proteins, and reduced the occupancy of the Menin–MLL1 fusion protein complex on the HoxA9 promoter resulting in hematopoietic differentiation.^102^

The Cierpicki lab also synthesized MI‐2‐2 with much higher affinity [*K*_D_ of 22 n*M*; IC_50_ (K_disp_) of 46 n*M*] through structure‐based follow up chemistry by replacing n‐propyl with a trifluoroethyl group in MI‐2.^111^ MI‐2‐2 disrupted the interaction of Menin and MLL1–AF9 in HEK293 cells at low micomolar concentrations, about fourfold more potent than MI‐2. Overall, MI‐2‐2 showed significantly higher cellular activity with 80% reduction of HoxA9 and Meis‐1 expression at 6 µ*M* and exhibited significant effect in human leukemia cell line MV4;11 carrying the MLL1–AF4 translocation, which is consistent with the improved potency towards the Menin–MLL1 interaction.^111^ He and colleagues^112^ also identified hydroxy‐ and aminomethylpiperidines as inhibitors of the Menin–MLL1 interaction through screening a library of 288,000 compounds by FP. The IC_50_ (*K*_disp_) for the best hit was 12.8 µ*M*. Follow‐up structure‐guided chemistry resulted in synthesizing MIV‐6R with IC_50_ (*K*_disp_) value of 56 n*M* that inhibited proliferation and induced hematopoietic differentiation in MLL1‐AF9, ‐AF6, and ‐AF1p fusion leukemia cells indicating behaviour independent of the fusion partner.^112^ Orally bioavailable derivatives of MI‐2‐2, MI‐503, and MI‐463 were developed that inhibited the growth of MLL1 fusion cell lines, induced differentiation and were effective in the xenograft models blocking leukemia progression.^113^

In a different approach, Zhou and colleagues used a linear MLL1 octameric peptide (MLL1 residues 6‐13; ‐RWRFPARP) as a starting point to develop macrocyclic peptidomimetic antagonists of the Menin–MLL interaction. These structure‐guided chemistry efforts resulted in design and synthesis of MCP‐1 (*K*_i_: 4.7 n*M*).^114^

### Other inhibitors affecting MLL‐mediated leukemogenesis

MLL1 and Menin bind to the genomic Hox loci to activate gene expression.^115^, ^116^ It has been reported that MLL1 fusion proteins may also promote gene expression by increasing the H3K79me2 mark in leukemia stem cells. Inactivation of DOT1L, the only known H3K79 methyltransferase, led to downregulation of direct MLL1–AF9 targets and an MLL translocation‐associated gene expression signature, while global gene expression remained largely unaffected. These data support DOT1L as a potential therapeutic target in MLL1‐rearranged leukemia.^117^ It has been reported that early mammalian erythropoiesis requires DOT1L activity. In early hematopoiesis, DOT1L regulates the expression of a critical differentiation switch that controls the numbers of circulating erythroid and myeloid cells.^118^ In recent years several potent SAM‐competitive inhibitors of DOT1L have been reported. EPZ004777 from Epizyme was the first reported potent DOT1L inhibitor that selectively suppressed leukemia cells with MLL1 translocation.^119^ A second DOT1L inhibitor, EPZ‐5676 was later discovered by Epizyme with higher potency, selectivity and better pharmacokinetics and is currently in phase I clinical trial.^120^, ^121^, ^122^ These compounds have been reviewed in more details by Chen and Armstrong.^21^ SGC0946, a brominated analog of EPZ004777 was later reported with enhanced potency and increased cellular activity over EPZ004777 likely due to its longer residence time on the protein.^95^ SYC‐522 was also reported as a potent DOT1L inhibitor.^123^

## Summary

Here we have summarized the multiple opportunities for targeting the SET1 family of proteins. First, we summarized the literature surrounding the characterization of these enzymes and their recombinant complexes suitable for small molecule screening, including new kinetic data from our lab. This body of data should facilitate efforts to find new inhibitors/modulators of enzymatic function of these proteins—a traditional approach to drug discovery. Interestingly, however, to‐date there is more progress in targeting the SET1 family by targeting the extensive network of protein–protein interactions involving wild‐type SET1 family proteins and/or oncogenic MLL1 fusion proteins. Fluorescence polarization‐based peptide displacement methods have proven to be efficient and cost effective for high‐throughput screening to identify antagonists of WDR5–MLL1 and Menin–MLL1 interactions. Highly potent and selective antagonists of such interactions have been shown to effectively disrupt the MLL1 complex with WDR5, and Menin and decrease the expression of HoxA9 and Meis‐1, inhibiting proliferation and inducing hematopoietic differentiation in MLL1 leukemia cells.

## Supporting information

Supporting Information
