# Supplementary material for: Targeting human SET1/MLL family of proteins
Source: Protein Sci. 2017 Mar 6;26(4):662–76. doi: 10.1002/pro.3129 (PMC5368065; doi:10.1002/pro.3129)
Supplement: Supplementary file 1 — Supporting Information [file PRO-26-662-s001.pdf]

Supplemental Information

Targeting human SET1/MLL family of proteins

Masoud Vedadi[1,2*], Levi Blazer[1], Mohammad S. Eram[1], Dalia Barsyte-Lovejoy[1], Cheryl H. Arrowsmith[1,3], and Taraneh Hajian[1]

[1]Structural Genomics Consortium, University of Toronto, Toronto, ON, M5G 1L7

[2]Department of Pharmacology and Toxicology, University of Toronto, Toronto, ON, M5S 1A8.

[3]Princess Margaret Cancer Centre and Department of Medical Biophysics, University of Toronto, Toronto, ON, M5G 2M9.

*To whom correspondence should be addressed:

Masoud Vedadi, Tel: (416) 432-1980; E-mail: m.vedadi@utoronto.ca

Supplemental Information Index:

Supplemental Materials and Methods 2-6

Table S1. Assay conditions for MLL family complexes 5

Figure S1. Optimization of trimeric SET1A and SET1B activity 6

Figure S2. Kinetic characterization of MLL1 7

Figure S3. Kinetic characterization of MLL3 8

Figure S4. Kinetic characterization of SET1A 9

Figure S5. Kinetic characterization of SET1B 10

Figure S6. Linearity of initial velocities 11

Figure S7. Assay optimization 15

Supplementary References 16

Supplemental Materials and Methods:

Chemicals

[^{3}H]S-adenosylmethionine was obtained from Perkin Elmer (Waltham, MA) and S-adenosylmethionine was obtained from AK Scientific (Union City, CA). S-adenosylhomocysteine was obtained from (Sigma). Biotinylated peptide substrates were obtained from Peptide2.0 (Chantilly, VA) or from Tufts University Peptide Synthesis Core Facility (Boston, Ma). Peptide sequences were as follows: H3 1-25: ARTKQTARKSTGGKAPRKQLATKAA-GK-biotin; H3K4me1: ART(Kme1)QTARKSTGGKAPRKQLATKAA-GK-biotin; H3K4me2: ART(Kme2)QTARKSTGGKAPRKQLATKAA-GK-biotin. Unless otherwise indicated, all other chemicals were obtained from Sigma or LifeTech and were of reagent grade or better.

Cloning and Protein Production:

Expression and purification of the MLL1 trimeric complex (MLL1:RbBP5:WDR5) and ASH2L were performed as previously described(1). Plasmids containing the cDNA for human SET1A (1491-1707), SET1B (1815-2037), and MLL3 (4706-4911) were generously provided by Dr. Jean-Francois Couture (University of Ottawa) and were cloned into the following vectors: pSMT2 (SETD1A); pSMT3 (SETD1B and MLL3). A prokaryotic expression plasmid for human DPY30 was generated by subcloning the cDNA for full-length DPY30 (1-99) into the pET28-MHL vector. A co-expression construct for RbBP5 (1-538) and WDR5 (1-334) were generated by sub-cloning the cDNA into a dual expression system based upon the pFastBac-Dual vector.

His-tagged RbBP5 (1-538) and WDR5 (1-334) were co-overexpressed in Sf9 cells grown in HyQ® SFX insect serum-free medium (TermoScientific). Cells were infected and incubated at 27 °C using a platform shaker set at 100 RPM for a period of 72 hours. Harvested cells were re-suspended in 20 mM Tris-HCl buffer, pH 7.5, containing 500 mM NaCl, 5 mM imidazole and 5% glycerol, 1X protease inhibitor cocktail (100 X protease inhibitor stock in 70% ethanol (0.25 mg/ml Aprotinin, 0.25 mg/ml Leupeptin, 0.25 mg/ml Pepstatin A and 0.25 mg/ml E-64) or Roche complete EDTA-free protease inhibitor cocktail tablet. The cells were lysed chemically

by rotating 30 min with NP40 (final concentration of 0.6%) and 50 U/mL Benzonase Nuclease (Sigma) and 2 mM 2-mercaptoethanol followed by sonication at frequency of 8 (10”on/10”off) for 4 min (Sonicator 3000, Misoni). The crude extract was clarified by high-speed centrifugation (60 min at 36,000 ×g at 4 ^{o}C). The recombinant protein complex was purified by loading the cleared lysate on a Talon metal affinity resin (Clontech) followed by washing with 20 mM Tris-HCl, pH 7.5, 500 mM NaCl, 5% glycerol, 15 mM imidazole). The recombinant protein complex was eluted from the cobalt-based affinity column using the elution buffer (20 mM Tris-HCl, pH 8.0, 500 mM NaCl, 5% glycerol, 250 mM imidazole). The eluate with 3 mM 2-mercaptoethanol and 1X Roche complete EDTA-free protease inhibitor was concentrated and loaded onto a gel filtration Superdex-200 column (GE Healthcare) and eluted with sodium phosphate buffer (50 mM, pH 7.0), 150 mM NaCl and 5 mM 2-Mercaptoethanol. The pure fractions (as judged by SDS–PAGE) were pooled, concentrated, and flash frozen.

Recombinant DPY-30 was overexpressed as a polyhistidine fusion protein in *E. coli* BL21 (DE3)-V2R-pRARE2 by addition of 1 mM IPTG and incubated overnight at 15 ˚C. Harvested cells were resuspended in 20 mM Tris-HCl, pH 7.5, 500 mM NaCl, 5 mM imidazole, and 5% glycerol, supplemented with 1X protease inhibitor cocktail (100X protease inhibitor stock in 70% ethanol (0.25 mg/ml Aprotinin, 0.25 mg/ml Leupeptin, 0.25 mg/ml Pepstatin A and 0.25 mg/ml E-64) or 1X Complete EDTA-free protease inhibitor cocktail tablet (Roche Applied Science, Penzberg, Germany). Cells were chemically lysed by rotating for 30 min with 0.5% CHAPS and 22.5 U/mL Benzonase nuclease followed by sonication at frequency of 8.5 with a 50% duty cycle for 4 min (Sonicator 3000, Misoni). The crude extract was clarified by high-speed centrifugation (60 min at 36,000 ×g at 4 ^{o}C) and the resulting clarified lysate was loaded onto a DE52 ion-exchange resin (GE Healthcare Life Sciences) and passed through a Hispur^{TM} Ni-NTA resin (Thermo Scientific) column. The column was washed with 20 mM Tris-HCl, pH 7.5, 500 mM NaCl, 5% glycerol, 30 mM imidazole and retained protein was eluted in the same buffer containing 250 mM imidazole. Purified DPY30 was dialyzed against 50 mM sodium phosphate pH 7.0, 150 mM NaCl and 5 mM 2-Mercaptoethanol and concentrated for storage. Purity was confirmed by SDS-PAGE and LC-MS.

SET domains of SET1A and SET1B were overexpressed as His-SUMO fusion in *E. coli* Arctic cell and MLL3 in E.coli Rossetta cell (Agilent Technologies, Santa Clara, CA) during an

overnight incubation at 18 ^{o}C in the presence of 1 mM isopropyl-1-thio-D-galactopyranoside (IPTG). Harvested cells were re-suspended in 50 mM sodium phosphate pH 7.0, 300 mM NaCl, 10% glycerol, and 1X protease inhibitor cocktail (100 X protease inhibitor stock in 70% ethanol (0.25 mg/ml Aprotinin, 0.25 mg/ml Leupeptin, 0.25 mg/ml Pepstatin A and 0.25 mg/ml E-64) or 1X Complete EDTA-free protease inhibitor cocktail tablet (Roche Applied Science, Penzberg, Germany). Cells were lysed chemically by rotating for 30 minutes in the presence of 0.6% NP40 and 3 mM 2-mercaptoethanol followed by sonication at a frequency of 8 with a 50% duty cycle for 3 min (Sonicator 3000, Misoni). Crude extract was clarified by high-speed centrifugation (60 min at 36,000 ×g at 4 ^{o}C). Recombinant protein loaded onto a Talon metal affinity resin (Clontech, Mountain View, CA) and washed with 10 column volumes of 50 mM sodium phosphate buffer pH 7.0, 300 mM NaCl, 10% glycerol and 5 mM 2-Mercaptoethanol. Untagged recombinant protein was liberated from the column by Sumo Protease in the same buffer for at least 2 hours at 4 ^{o}C and the untagged SET domain protein was recovered.

MLL complexes were formed immediately after purification of the SET domain by mixing the desired protein members in equimolar ratios during a 2-hour dialysis at 4 ^{o}C against Bis-tris propane (20 mM, pH 7.0), 250 mM NaCl and 5 mM 2-mercaptoethanol. The complexes were concentrated and purified over a Superdex-200 16/60 size exclusion column in the same buffer. Complex order and purity was determined using SDS-PAGE analysis.

Histone methyltransferase assays

Histone methyltransferase assays were performed in an assay buffer consisting of 20 mM Tris-HCl pH 8.0, 0.01% Triton X-100, and 5 mM DTT. All MLL complexes were desalted using Zeba spin columns into assay buffer before use (Thermo Fisher, Cat. 89882). Reactions were prepared by pre-mixing a methyltransferase complex with a histone peptide and initiated by the addition of [^{3}H] S-adenosylmethionine [PerkinElmer Life Sciences, cat # NET155V001MC; specific activity range 12−18 Ci/mmol] in a total reaction volume of 10 µl. Reactions were allowed to proceed at 23 °C before being quenched with excess guanidinium hydrochloride. Quenched reactions were spotted on SAM2$^{©}$ Biotin Capture Membranes (Promega, Madison, WI). Membranes were washed twice with 2 M NaCl, twice with water, and then allowed to air dry before retained radioactivity was measured using liquid scintillation analysis in a Tri-Carb

liquid scintillation counter (Perkin Elmer, Waltham MA). Assay conditions are as described in the table below.

Table S1. Assay conditions for MLL family complexes.

Protein	Complex	Enzyme concentration (nM)	Reaction Time (min)	SAM saturation for peptide kinetics (µM)	Peptide saturation for SAM kinetics (µM)
MLL1	Trimeric	500	30	50	25
	Tetrameric	100	30	20	25
	Pentameric	100	20	10	10
MLL3	Trimeric	500	60	250	100
	Tetrameric	50	15	250	50
	Pentameric	50	15	250	50
SET1A	Trimeric	500*	60	500	100
	Tetrameric	100	60	500	100
	Pentameric	100	60	500	100
SET1B	Trimeric	500*	10	50	25
	Tetrameric	100	60	50	25
	Pentameric	100	30	50	25

*Concentration of SET domain of SET1A and SET1B within the trimeric complex was increased to molar ratio of 4:1.

While trimeric SET1A and SET1B eluted from the gel filtration column as a complex, no activity was observed with the peptide substrate. However, increasing SET domain / WDR5-RbBP5 ratio to 4:1 instead of 1:1 resulted in active trimeric complex. For these experiments, 500 nM of the trimeric complex was mixed with various amounts of additional purified SET domain for 10 minutes at room temperature before performing activity assays. SET1A reactions were performed for 60 minutes in the presence of 500 µM SAM and 100 µM H3 1-25 biotin peptide, while SET1B reactions were performed for 60 minutes in the presence of 50 µM SAM and 25 µM H3 1-25 biotin peptide. Incorporated radioactivity was quantified using biotin capture membranes as described above. It was determined that maximal activity for trimeric complexes was obtained at a 4x molar ratio of SET domain to previously prepared trimeric complex and this mixture was used for the determination of kinetic parameters.

Kinetic constants were calculated using nonlinear least squares regression analysis in GraphPad Prism v. 6.03 using the Michaelis-Menten model. For instances in which regression analysis

produced unreliable fits, the data are reported as greater than the highest concentration of substrate utilized in the experiment.

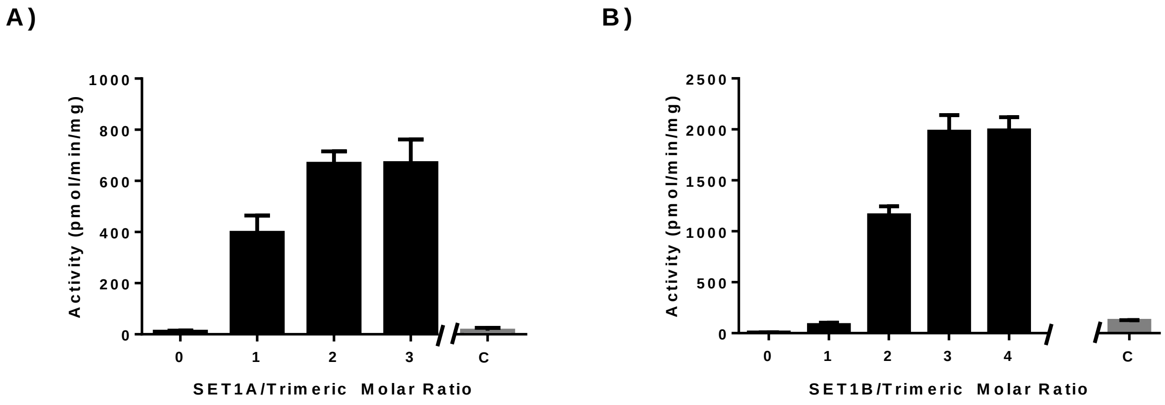

Figure S1. Optimization of trimeric SET1A and SET1B activity by increasing SET domain to WDR5-RbBP5 ratio. A) Trimeric SET1A (500 nM) was incubated with increasing amounts of additional SET1A SET domain before determination of methyltransferase activity as described in *Materials and Methods*. The molar ratios of SET1A SET domain to WDR5-RbBP5 complex are as follows: 0 (1:1), 1 (1:2), 2 (1:3) and 3 (1:4). B) Trimeric SET1B (500 nM) was also incubated with increasing amounts of additional SET1B SET domain before determination of methyltransferase activity. The molar ratios of SET1B SET domain to WDR5-RbBP5 complex are as follows: 0 (1:1), 1 (1:2), 2 (1:3), 3 (1:4) and 4 (1:5). Control (C) is 1500 nM of SET1A and SET1B alone.

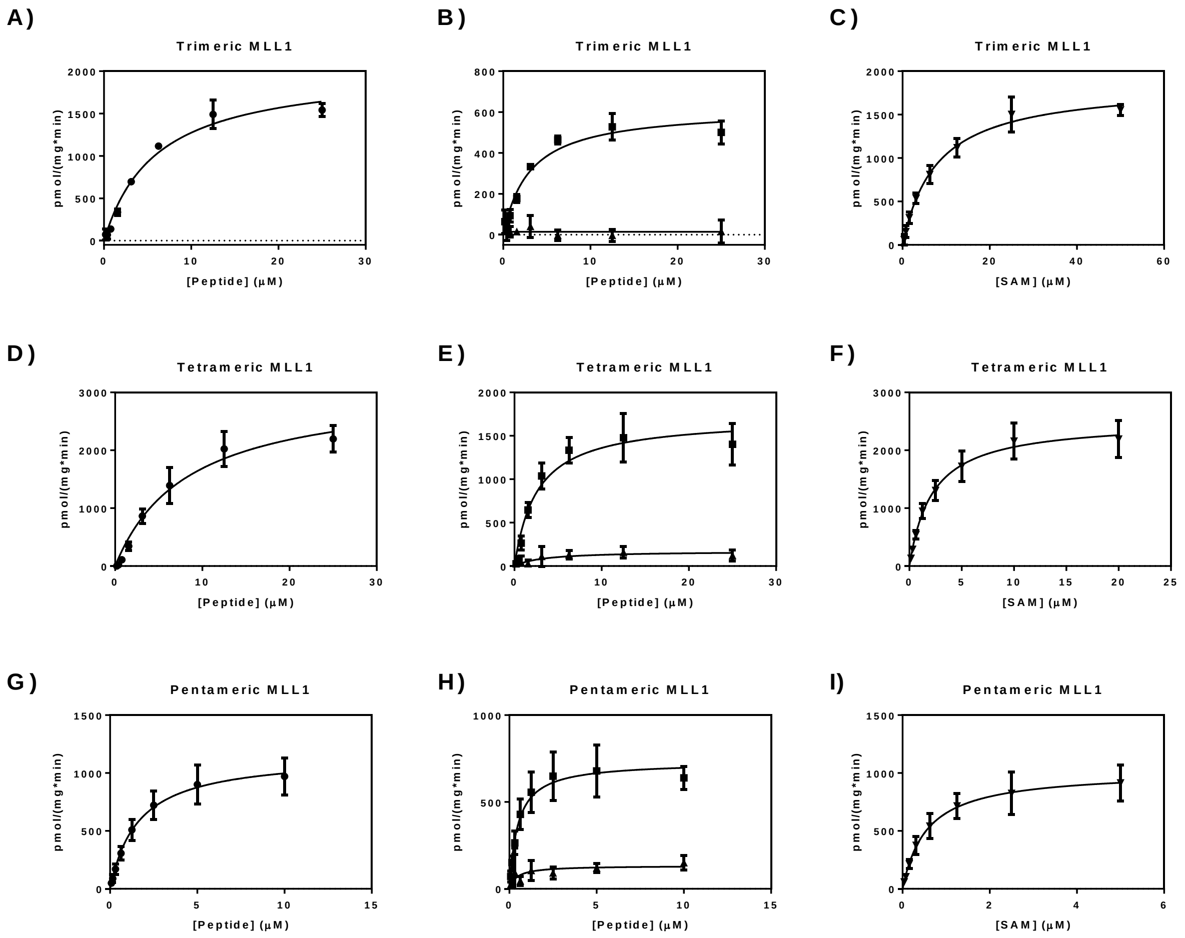

Figure S2. Kinetic characterization of MLL1 complex. Methylation of histone H3 1-25 peptide by A) trimeric, D) tetrameric, or G) pentameric MLL1 was assessed. Methylation of monomethylated (■) or dimethylated (▲) H3 1-25 peptide at lysine 4 by B) trimeric, E) tetrameric, or H) pentameric MLL1 complexes were also evaluated. SAM utilization by C) trimeric, F) tetrameric or I) pentameric MLL1 complexes in the methylation of histone H3 1-25 peptide substrate are shown. Kinetic constants derived from these data are presented in Table 1. Data are presented as the mean ± SD from three independent experiments.

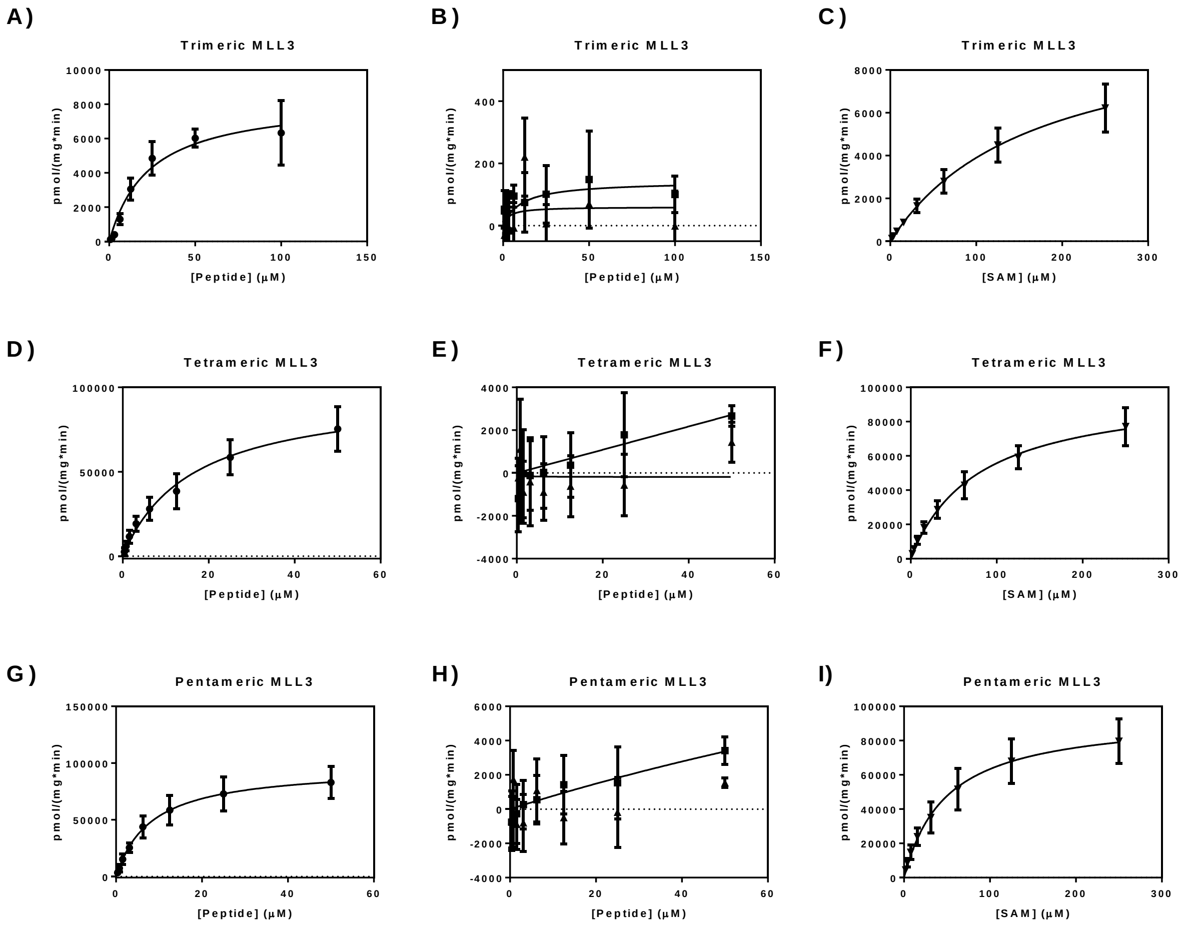

Figure S3. Kinetic characterization of MLL3 complexes. Methylation of histone H3 1-25 peptide by A) trimeric, D) tetrameric, or G) pentameric MLL3 was assessed. Methylation of monomethylated (■) or dimethylated (▲) H3 1-25 peptide at lysine 4 by B) trimeric, E) tetrameric or H) pentameric MLL3 were also evaluated. SAM utilization by C) trimeric, F) tetrameric or I) pentameric MLL3 complexes in the methylation of histone H3 1-25 peptide substrate are shown. Kinetic constants derived from these data are presented in Table 1. Data are presented as the mean ± SD from three independent experiments.

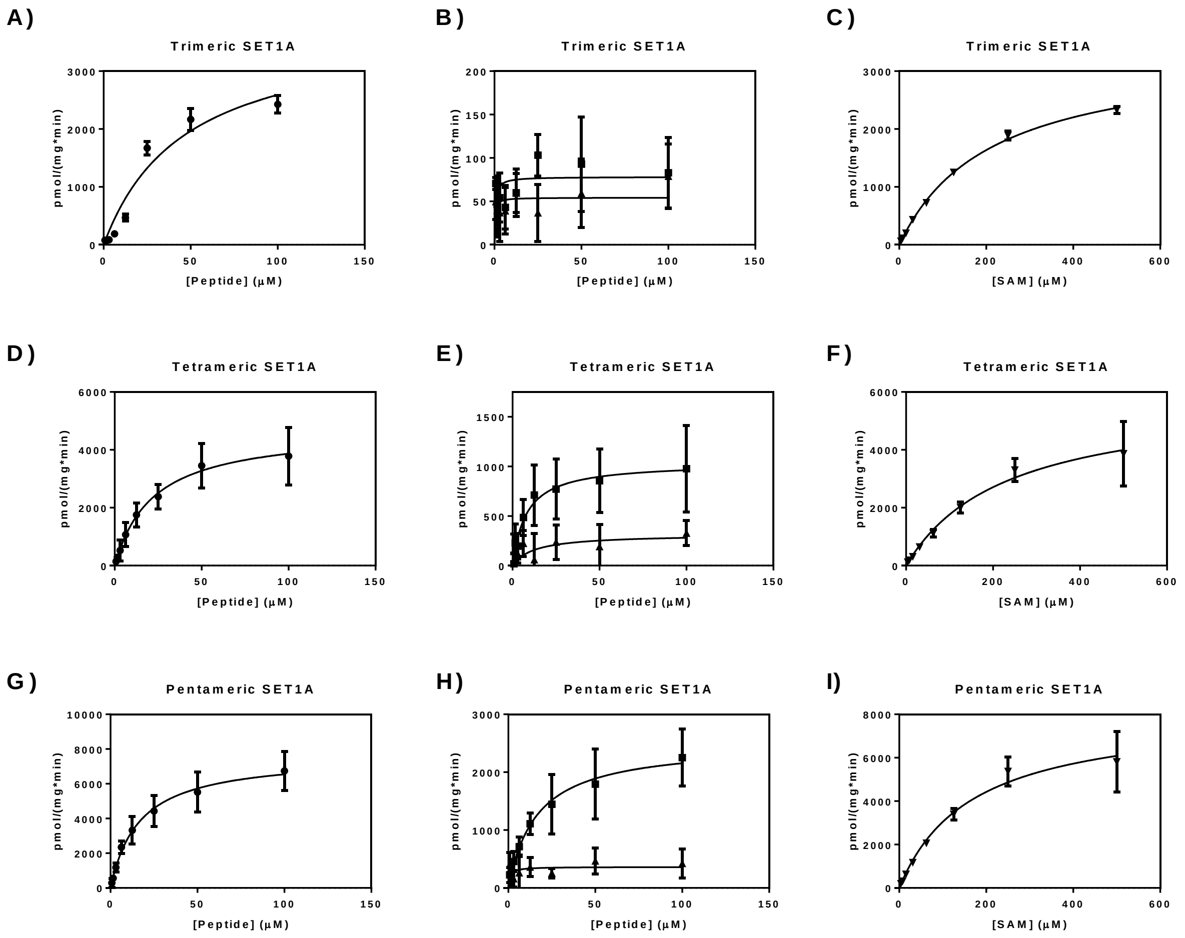

Figure S4. Kinetic characterization of SET1A complexes. Methylation of histone H3 1-25 peptide by A) trimeric, D) tetrameric, or G) pentameric SET1A was assessed. Methylation of monomethylated (■) or dimethylated (▲) H3 1-25 peptide at lysine 4 by B) trimeric, E) tetrameric, or H) pentameric SET1A were evaluated. SAM utilization by C) trimeric, F) tetrameric or I) pentameric SET1A in the methylation of histone H3 1-25 peptide substrate are shown. Kinetic constants derived from these data are presented in Table 1. Data are presented as the mean ± SD from three independent experiments.

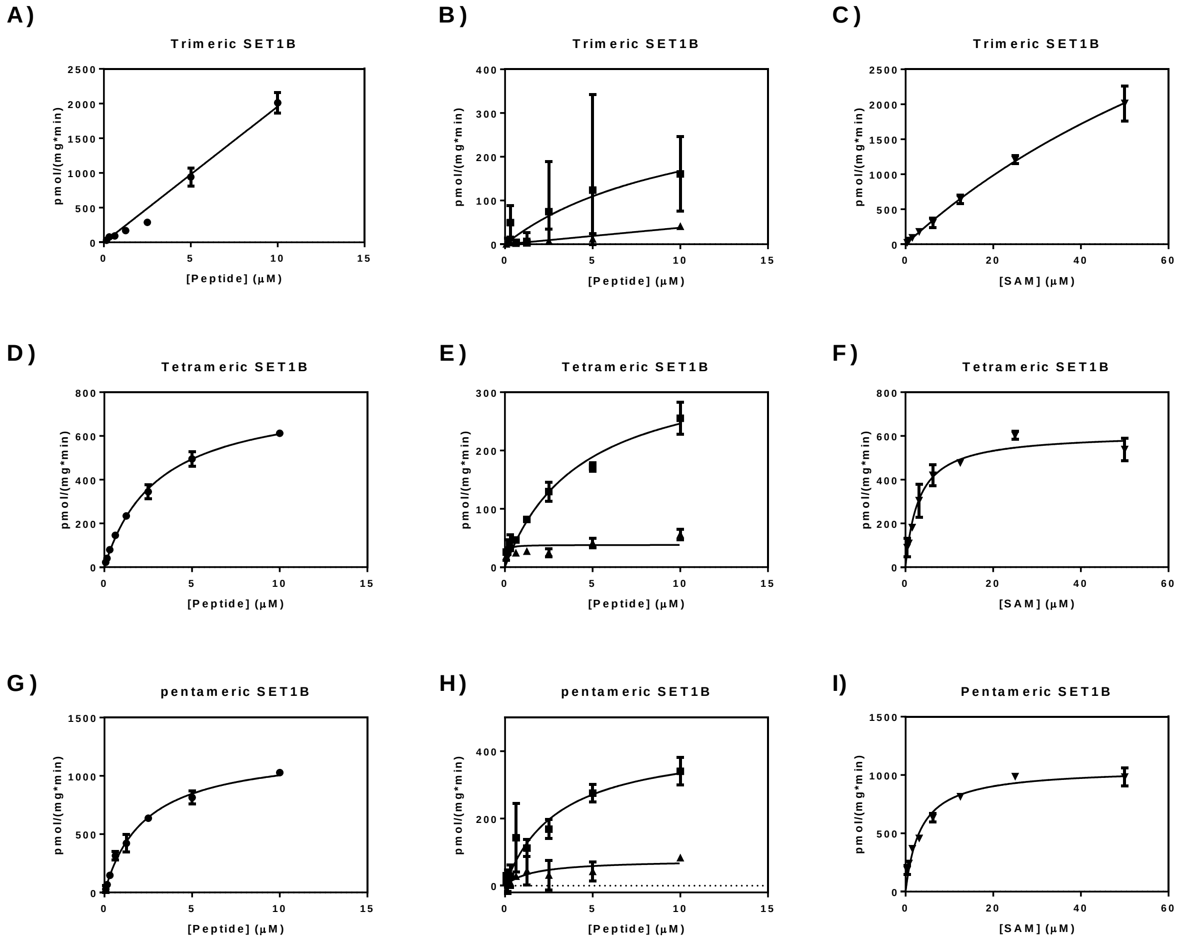

Figure S5. Kinetic characterization of SET1B complexes. Methylation of histone H3 1-25 peptide by A) trimeric, D) tetrameric, or G) pentameric SET1B was assessed. Methylation of monomethylated (■) or dimethylated (▲) H3 1-25 peptide at lysine 4 by B) trimeric, E) tetrameric, or H) pentameric SET1B was evaluated. SAM utilization by C) trimeric, F) tetrameric or I) pentameric SET1B in the methylation of histone H3 1-25 peptide substrate are shown. Kinetic constants derived from these data are presented in Table 1. Data are presented as the mean ± SD from three independent experiments.

Figure S6 (A); MLL1

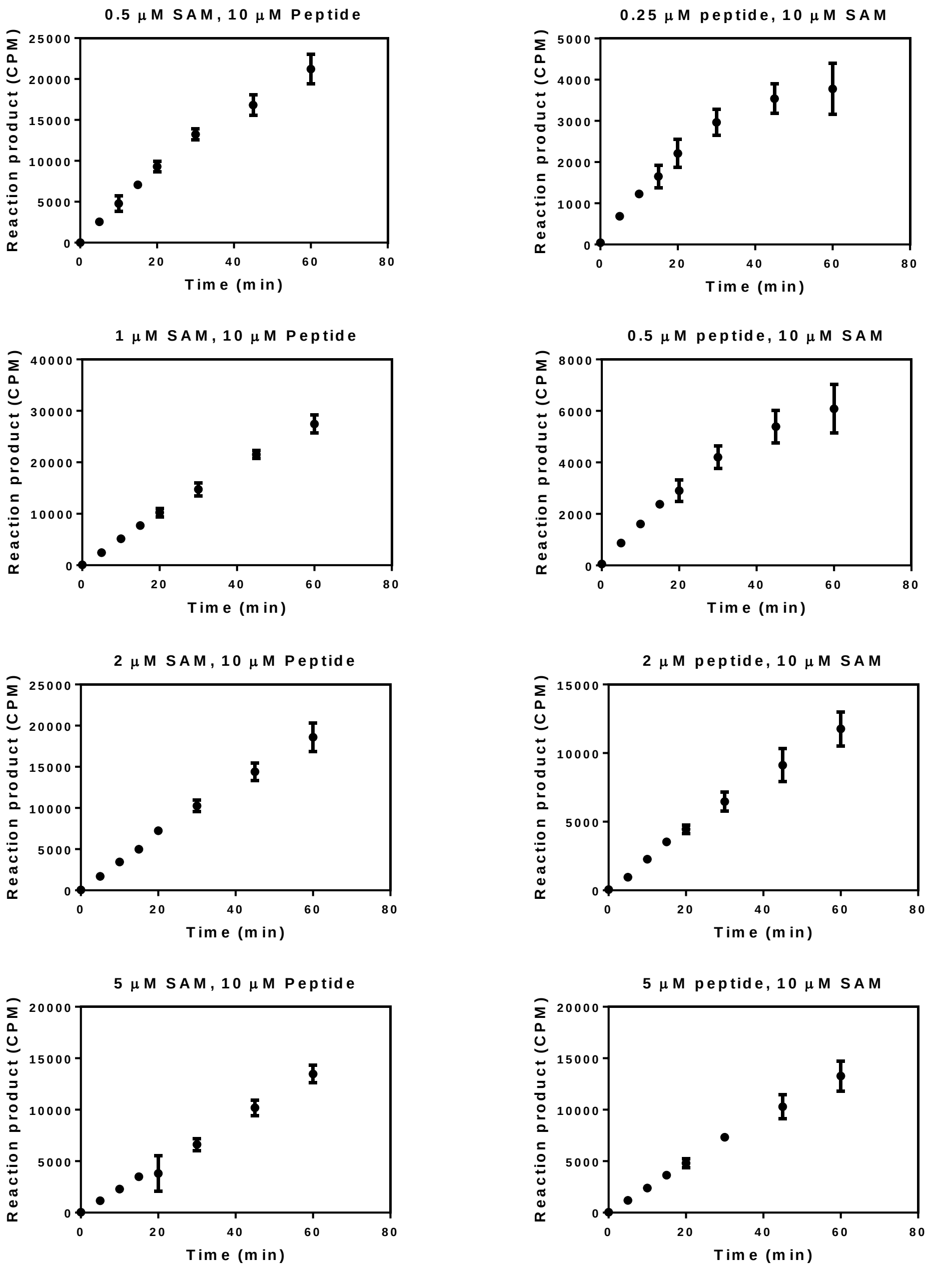

Figure S6 (B); MLL3

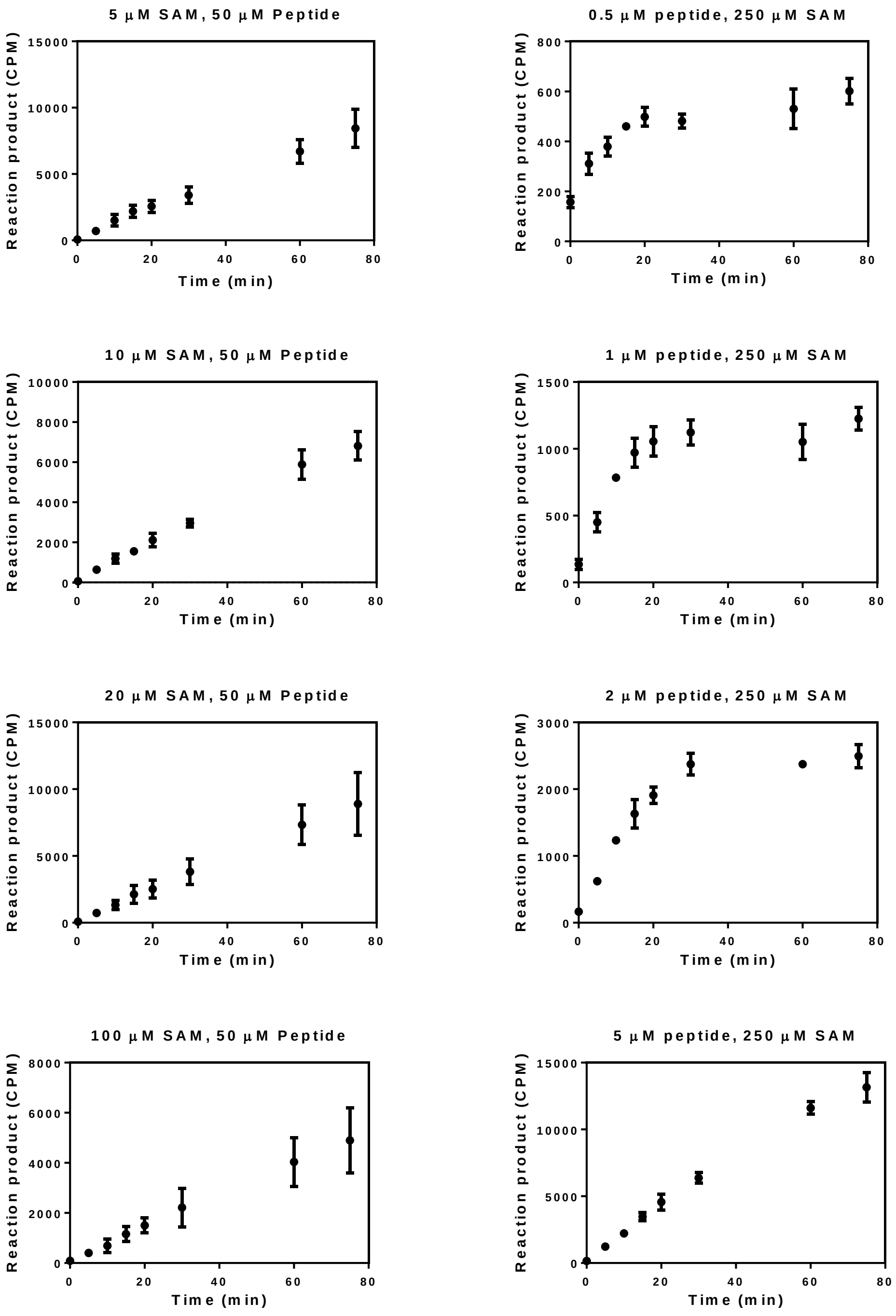

Figure S6 (C); SET1A

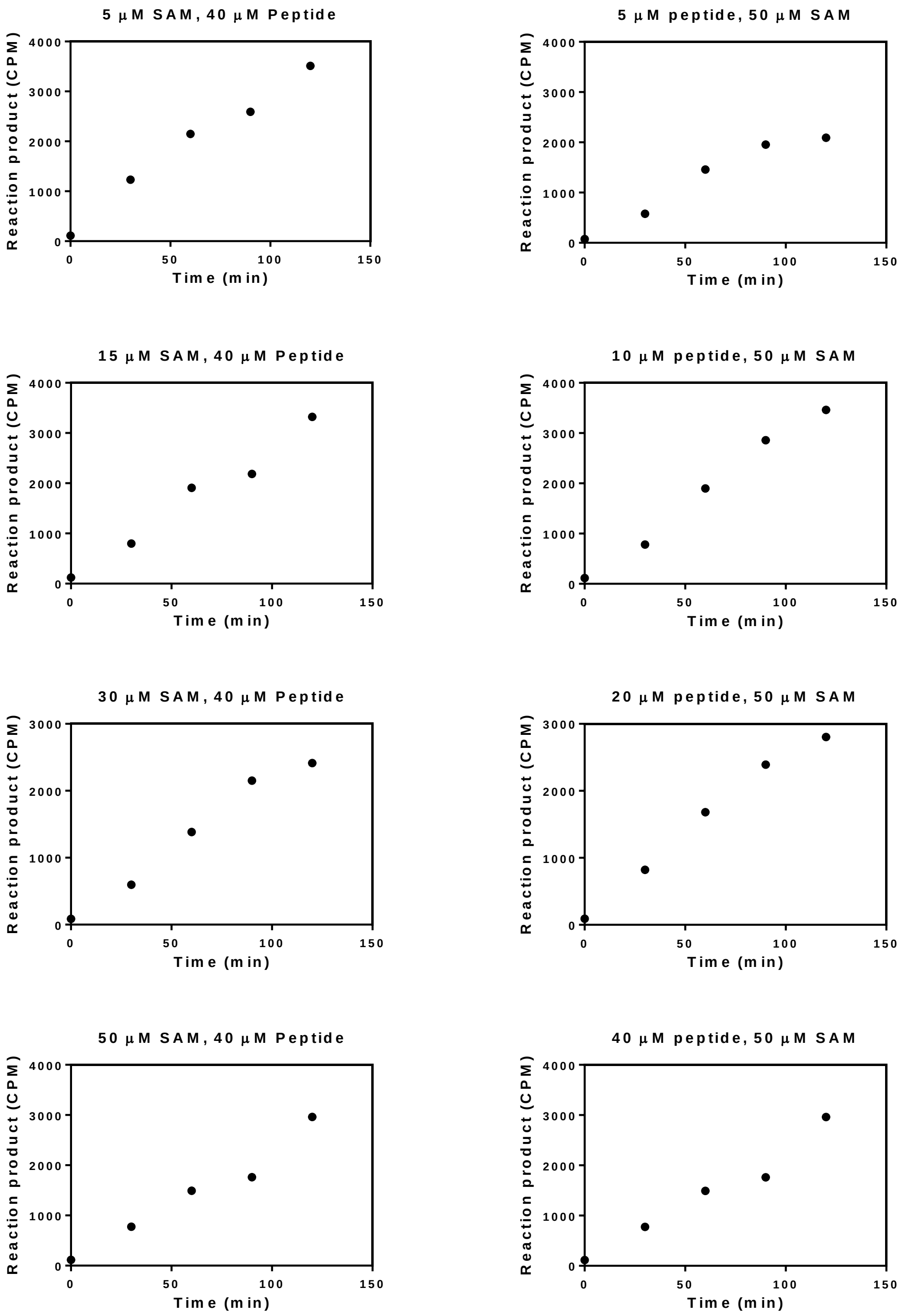

Figure S6 (D); SET1B

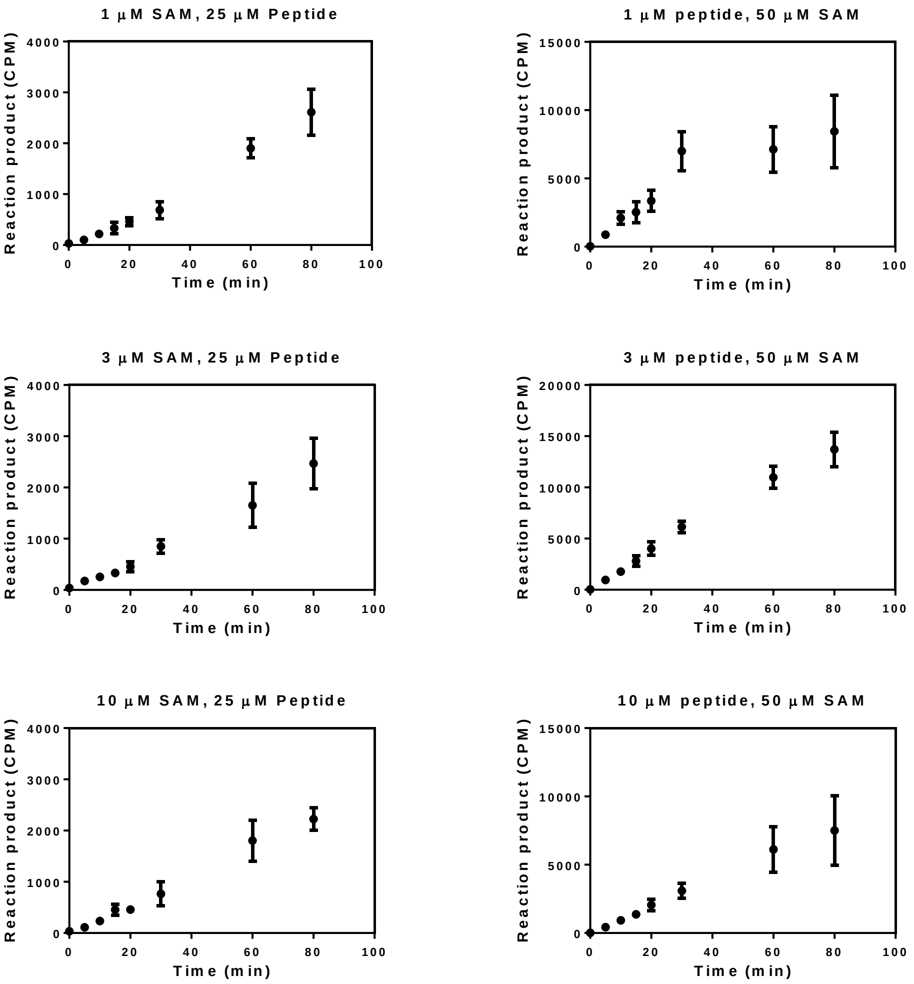

Figure S6. Linearity of the initial velocities. The linearity of initial velocities were confirmed for pentameric complexes of A) MLL1, B) MLL3, C) SET1A, and D) SET1B at varying concentrations of one substrate and saturating concentrations of the second substrate as indicated for each plot. Reaction time for performing experiments to determine kinetic parameters in each case was chosen according to linearity of initial velocities.

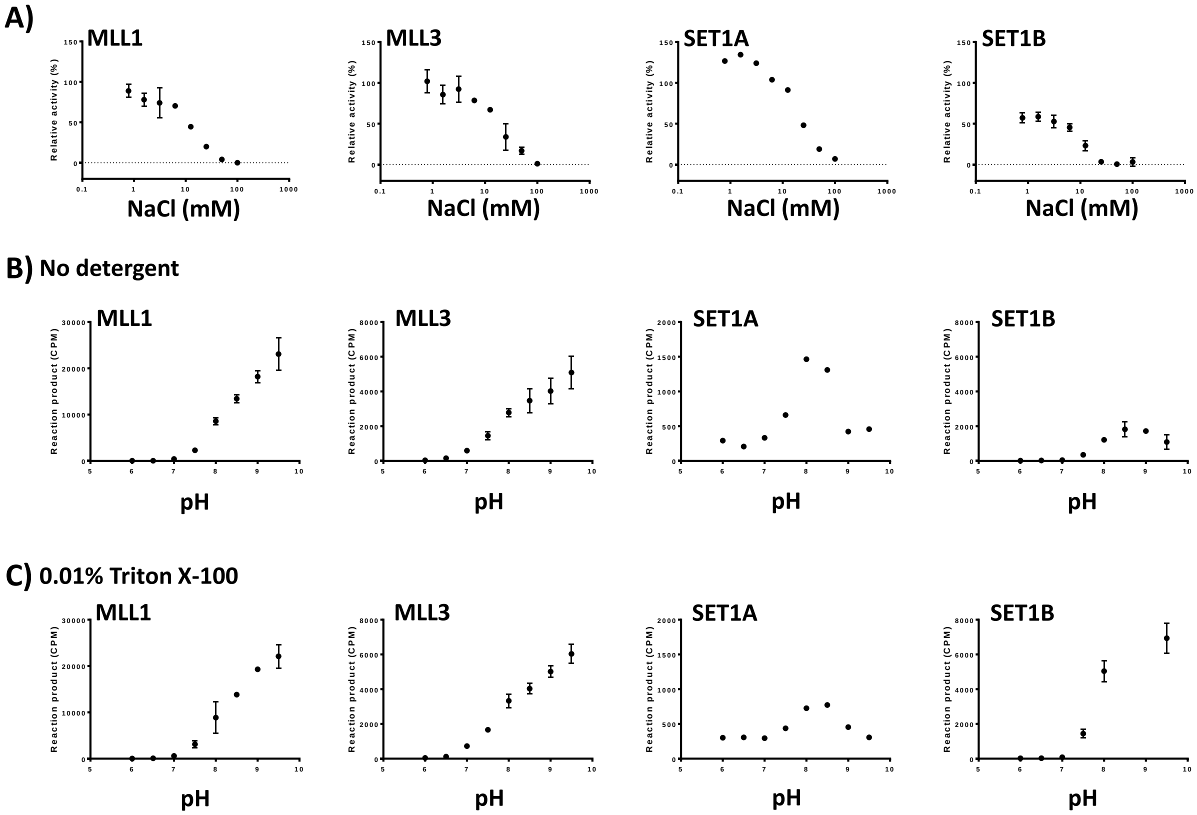

Figure S7. Effects of salt, pH and detergents on activity of SET1 family of proteins. (A) The effects of sodium chloride (NaCl) on activity of pentameric complexes of MLL1, MLL3, SET1A, and SET1B were evaluated. NaCl at concentrations as low as 10 mM significantly reduced the activity of all four pentameric complexes as specified. pH profile for each enzyme was determined B) in the absence and C) presence of 0.01% Triton X-100.

Supplementary References

1. Senisterra G, Wu H, Allali-Hassani A, Wasney GA, Barsyte-Lovejoy D, Dombrovski L, Dong A, Nguyen KT, Smil D, Bolshan Y, Hajian T, He H, Seitova A, Chau I, Li F, Poda G, Couture JF, Brown PJ, Al-Awar R, Schapira M, Arrowsmith CH, Vedadi M (2013) Small-molecule inhibition of MLL activity by disruption of its interaction with WDR5. The Biochemical journal 449:151-159. PMID: 22989411
